# Transcriptomic Comparison of Soybean Roots Inoculated with Different Rhizobium Strains During Early Symbiosis

**DOI:** 10.3390/plants15091417

**Published:** 2026-05-06

**Authors:** Qin Lin, Ziji Wu, Ruixin Xu, Jing Zhang, Min Deng, Tao Wang, Qi Zhang, Peiwu Li, Zhe Yan

**Affiliations:** 1 Oil Crops Research Institute, Chinese Academy of Agricultural Sciences, Wuhan 430062, China; linqin2022@126.com (Q.L.); zhangqi01@caas.cn (Q.Z.); 2 Xianghu Laboratory, Hangzhou 311231, China; wuziji1020@163.com (Z.W.); zj18756563856@163.com (J.Z.); dengmin@xhlab.ac.cn (M.D.); wangtao@xhlab.ac.cn (T.W.); 3 The National Key Facility for Crop Gene Resources and Genetic Improvement (NFCRI)/State Key Laboratory of Crop Gene Resources and Breeding/Key Laboratory of Crop Gene Resource and Germplasm Enhancement (MOA)/Key Laboratory of Grain Crop Genetic Resources Evaluation and Utilization, Institute of Crop Sciences, Chinese Academy of Agricultural Sciences, Beijing 100081, China; xuruixin@iga.ac.cn

**Keywords:** soybean (*Glycine max*), *B. ottawaense* Bott 59, symbiotic nitrogen fixation, transcriptome, isoflavonoid biosynthesis, phenylpropanoid biosynthesis

## Abstract

The symbiotic relationship between soybean and rhizobia facilitates nodulation and nitrogen fixation, providing a sustainable nutrient supply for increasing crop yields and reducing chemical fertilizer use. However, comparative studies on the conservation and strain-specificity of host gene expression regulated by different rhizobial strains remain limited. Here, we performed a comparative analysis between the previously isolated strain, *Bradyrhizobium ottawaense* Bott 59, and the model strain, *Bradyrhizobium diazoefficiens* USDA 110. Symbiotic phenotypes were evaluated after inoculation, and a root transcriptomic analysis was conducted at 3 dpi to assess early molecular responses. At 21 dpi, Bott 59-inoculated plants outperformed plants inoculated with USDA 110 in nodule number, nitrogenase activity, and biomass. Transcriptomic analysis revealed conserved host responses to both rhizobial strains, including NIN-mediated signaling, AON signaling, and the biosynthesis of phenylpropanoids and brassinosteroids. Further analysis revealed that Bott 59 specifically induced the expression of genes involved in isoflavonoid and flavonoid biosynthesis, including those encoding *I2H*, and *HI4OMT*. Moreover, Bott 59 triggered more pronounced transcriptional reprogramming in auxin, cytokinin, and jasmonic acid signaling pathways, along with differential expression of a broader set of transcription factor genes. Collectively, this study systematically unravels the conserved and strain-specific transcriptional regulatory events underlying host–rhizobium interactions. Our findings provide valuable theoretical insights and transcriptomic resources for further dissecting the molecular mechanisms of symbiotic nitrogen fixation (SNF), as well as for the targeted genetic improvement of crop nodulation and nitrogen fixation efficiency.

## 1. Introduction

Soybean (*Glycine max* L. Merr.) is a vital food and oil crop globally, providing high-quality protein and oil for human diets and serving as an excellent animal feed source [1]. With the rapid growth of the global population, increasing soybean productivity has become a priority in sustainable agriculture. Legumes have evolved to form specific symbiotic relationships with soil-borne rhizobia, leading to the development of nodules. Within these nodules, the nitrogenase system reduces atmospheric nitrogen (N_2_) into ammonia (NH_3_), which can be directly assimilated and utilized by the host plant. Nodules are also the central hub for molecular signaling and nutrient exchange between soybean and rhizobia [2]. Through this symbiotic nitrogen fixation (SNF) capability, legumes such as soybean are able to efficiently acquire the nitrogen essential for their growth and development even in low-nitrogen environments [3]. Legume–rhizobia symbiosis significantly enhances nitrogen use efficiency within ecosystems [4]. Globally, SNF in legume crops was estimated at 35.5 million tons in 2018, with soybean contributing the largest share at 25 million tons [5]. Beyond improving nitrogen acquisition, optimizing SNF efficiency has been shown to directly increase soybean yield [6]. Consequently, elucidating the molecular mechanisms governing SNF and further optimizing its efficiency are key technological pathways to boost soybean production, mitigate agricultural non-point source pollution, and promote sustainable green agriculture.

Nodule formation is initiated by a complex molecular dialog between the host legume and rhizobia: soybean roots release flavonoid signaling molecules that attract rhizobia and stimulate the secretion of Nod factors (NFs) [7,8,9]. Perception of NFs by the host plant promotes rhizobial attachment to root hairs, induces root hair curling, traps bacteria, and facilities the formation of infection threads (ITs) [10]. Rhizobia migrate through ITs to reach and colonize nodule primordia, where they ultimately develop into mature, nitrogen-fixing nodules [2,10,11]. Molecular mechanistic analyses have demonstrated that at the onset of symbiosis, NFs are perceived by LysM-type receptor-like kinases (LYKs) on the host cell membrane, specifically LjNFR1/MtLYK3 and LjNFR5/MtNFP [12]. RINRK1 directly interacts with the extracellular domains of NFR1/NFR5, amplifying the Nod factor signal [13]. This recognition event triggers sustained perinuclear and nuclear calcium oscillation [14,15]. This calcium signal is decoded by CCaMK, which subsequently activates the core symbiotic regulatory gene, like *NIN* and other downstream transcription factors, including *ERN*, *ARF*, *NSP1/2*, and *NLP*, ultimately controlling infection thread formation and nodule primordium development [16,17,18,19]. To coordinate resource allocation between plant growth and SNF, legumes have evolved the Autoregulation of Nodulation (AON) pathway. In this system, root-derived signaling peptides [20] are transported long-distance to shoots, where they are recognized by receptor kinases like NARK. This activates downstream signaling, inducing the production of molecules like miR2111 that are transported back to roots, ultimately fine-tuning nodule numbers negatively by regulating key genes such as *TML* [20,21,22].

Flavonoids are crucial signaling molecules during the establishment of legume–rhizobia symbiosis [23]. Isoflavonoids and flavonoids share the upstream biosynthetic pathway starting from phenylalanine to naringenin chalcone, catalyzed sequentially by phenylalanine ammonia-lyase (PAL), cinnamate-4-hydroxylase (C4H), 4-coumarate-CoA ligase (4CL), and chalcone synthase (CHS) [24]. In soybean, daidzein and genistein are the two predominant isoflavonoid components. Both are synthesized by isoflavone synthase (IFS) from flavanone substrates and play indispensable roles in nodule formation and the regulation of nodule number [25,26,27]. As a key branch of phenylpropanoid metabolism, the expression of isoflavonoid biosynthetic genes significantly impacts nodulation. Overexpressing isoflavonoid-related malonyl-CoA genes *GmMaT2* and *GmMaT4* promotes nodulation in the host [23,28]. Furthermore, several genes in the phenylpropanoid pathway show spatial expression specificity in mature *L. japonicus* nodules, suggesting that phenylpropanoid compounds may possess novel physiological functions during SNF [29]. CHS is a rate-limiting enzyme in flavonoid biosynthesis, and its expression directly impacts isoflavonoid production, and interfering with or silencing *MtCHS* genes leads to abnormal flavonoid accumulation in roots and significantly reduces nodule numbers [30,31]. Downregulation of *HCT* in alfalfa resulted in stunted root growth but unexpectedly increased nodule numbers, correlating with elevated levels of specific flavonoids [32]. Crucially, flavonoids with distinct structures act as chemoattractants for different rhizobia species and activate the expression of species-specific nodulation (*nod*) genes, underscoring their role as decisive factors in the symbiosis [33].

Nodule organogenesis is precisely regulated by a complex network of plant hormones. Cytokinin and auxin are core hormones that coordinately govern rhizobial infection and the initiation and development of nodules [34,35,36]. In terms of auxin regulation, overexpression of the auxin receptor genes *GmTIR1/GmAFB3* in soybean significantly increases infection events and total nodule number [37]. miR167 positively regulates soybean nodulation by targeting the transcription factor GmARF8 [16]. Flavonoids can inhibit polar auxin transport, leading to local auxin accumulation in roots, which creates a conducive hormonal environment for nodule primordium initiation and formation [38]. Cytokinin-induced nodule primordium formation is independent of the NF signaling pathway but depends on key regulators like *NIN*, *NSP1/2*, and *LHK1*. RNA interference (RNAi) of the cytokinin receptor homolog *MtCRE1* results in cytokinin insensitivity, exhibiting increased lateral root and significantly reduced nodulation [39]. This study also showed that *MtRR1* (type-A) and *MtRR4* (type-B), dependent on Nod factor signaling, are induced early during rhizobial infection, and activation of the cytokinin pathway positively regulates nodule formation [38,39,40]. Salicylic acid (SA) and jasmonic acid (JA) are involved not only in systemic acquired resistance but also in legume–rhizobia symbiosis. Reduced SA levels decreased the formation of ITs and nodule formation in *Lotus japonicus* but had the opposite effect in *Medicago truncatula* [41,42]. Jasmonates can induce *nod* gene expression in rhizobia [43]. However, the regulatory effect of JA on symbiosis is concentration-dependent, with studies showing both positive and negative roles depending on the concentration applied [38,41,42]. Other hormones like gibberellin are also involved, with the core regulator MtDELLA acting as a positive factor. *Mtdella* mutants exhibit severe defects in infection thread formation and nodule development. DELLA proteins also promote the formation of the CCaMK-IPD3 complex and interact with the NSP2-NSP1 transcriptional complex to significantly enhance the expression of Nod factor-induced genes, thereby driving nodule formation [44].

The biological process of SNF in legumes involves a complex gene regulatory network. Previous studies characterizing the omics data and molecular mechanisms of soybean SNF have predominantly utilized the model rhizobial strain *B. diazoefficiens* USDA 110 [23,28,37,45]. However, there remains a significant knowledge gap regarding the conserved and specific regulatory pathways induced by different rhizobial strains. In our previous work, we isolated a soybean rhizobial strain from Inner Mongolia, China, identified as *B. ottawaense* and designated Bott 59, which demonstrated excellent nodulation efficiency [46]. In the present study, we employed transcriptomics to compare the early root transcriptional profiles of soybean inoculated with Bott 59 and USDA 110. This study aims to elucidate the conserved mechanisms of soybean symbiotic signaling by identifying shared differentially expressed genes (DEGs) and to dissect the specific regulatory networks associated with the high-efficiency symbiosis of Bott 59 by comparing its unique DEGs with those of USDA 110. The findings of this research will provide insights into the molecular basis of efficient growth promotion by Bott 59, and identify potential targets for the molecular improvement of soybean SNF efficiency.

## 2. Results

### 2.1. Comparative Analysis of Symbiotic Performance Between Bott 59 and USDA 110 in Williams 82

**Figure 1:**
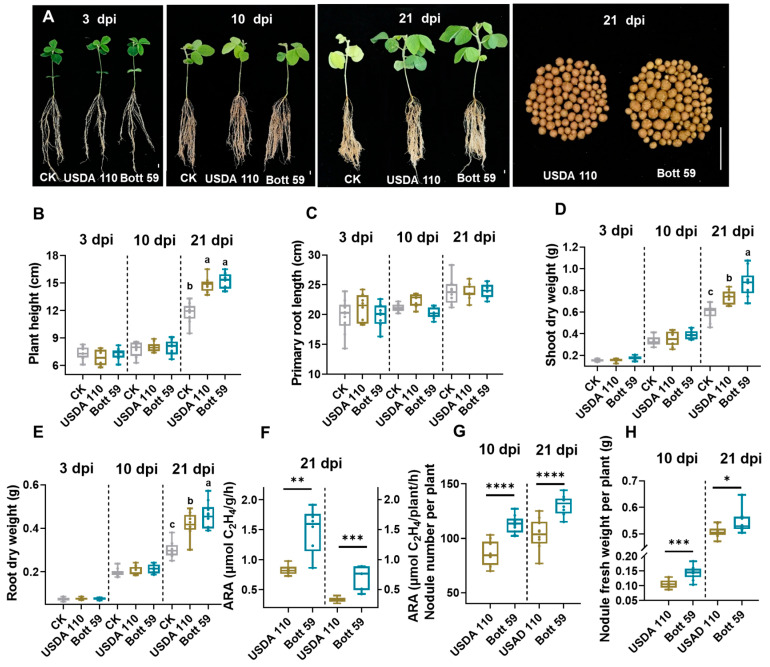
Inoculation with USDA 110 and Bott 59 promotes growth, nodulation, and nitrogen fixation in soybean cv. Williams 82. (**A**) Growth performance of soybean plants and nodules at various time points post-inoculation under inoculation with rhizobia or control (CK). After an initial 10-day cultivation, plant phenotypes are shown at 3, 10, and 21 dpi with or without rhizobia incubation. Nodule development is shown at 21 dpi for USDA 110 and Bott 59 inoculations. Scale bar = 1 cm. (**B**–**E**) Plant height (**B**), primary root length (**C**), shoot dry weight (**D**), and root dry weight (**E**) at 3, 10, and 21 dpi. (**F**) Acetylene reduction activity (ARA) of Williams 82 nodules at 21 dpi with USDA 110 and Bott 59 incubation. The left y-axis represents specific nitrogenase activity per nodule mass (µmol C_2_H_4_ g^−1^ h^−1^), and the right y-axis represents total nitrogenase activity per plant (µmol C_2_H_4_ plant^−1^ h^−1^). The two axes are separated by a vertical dashed line. (**G**,**H**) Nodule number per plant (**G**) and nodule fresh weight per plant (**H**) at 10 and 21 dpi. Boxes represent first quartile, median, and third quartile. Whiskers indicate minimum and maximum values; dots represent individual data points (*n* = 10, except for ARA, where *n* = 6). Different letters indicate statistically significant differences determined by one-way ANOVA followed by the Student–Newman–Keuls (SNK) post hoc test for multiple comparisons. All tests were two-tailed, with significance set at *p* < 0.05. Significant differences between USDA 110 and Bott 59 were determined by a two-tailed Student’s *t*-test (* *p* ≤ 0.05; ** *p* ≤ 0.01; *** *p* ≤ 0.001; **** *p* ≤ 0.0001).

To characterize the differential effects of rhizobial strains on soybean symbiosis and growth, we compared a newly isolated strain, *B. ottawaense* Bott 59 [46], to the conventional model strain, *B. diazoefficiens* USDA 110. Specifically, Williams 82 was inoculated with either Bott 59 or USDA 110 under greenhouse conditions. Plant growth and nodulation phenotypes were characterized at 3, 10, and 21 days post-inoculation (dpi) (Figure 1A). The results indicate that at 3 and 10 dpi, no significant differences were observed in plant height, primary root length, shoot dry weight, or root dry weight compared to the control (CK) (Figure 1A–E). However, by 21 dpi, both Bott 59- and USDA 110-inoculated groups displayed increased plant height, shoot dry weight, and root dry weight compared to the CK group (Figure 1B,D,E). Notably, when comparing the two strains, plants inoculated with Bott 59 exhibited higher shoot and root dry weights than those inoculated with USDA 110 at 21 dpi (Figure 1D,E). Moreover, at both 10 and 21 dpi, the nodule number and fresh nodule weight were greater in Bott 59- than in USDA 110-inoculated plants (Figure 1G,H). Additionally, the nitrogenase activity (ARA) was higher in Bott 59-inoculated plants than in the USDA 110-inoculated plants at 21 dpi (Figure 1F). In summary, compared to the conventional strain USDA 110, inoculation with the strain Bott 59 appears to promote the growth performance and SNF capacity.

### 2.2. Transcriptome Analysis of the Similarities and Differences in Host Gene Expression Regulated by Two Rhizobial Strains

**Figure 2:**
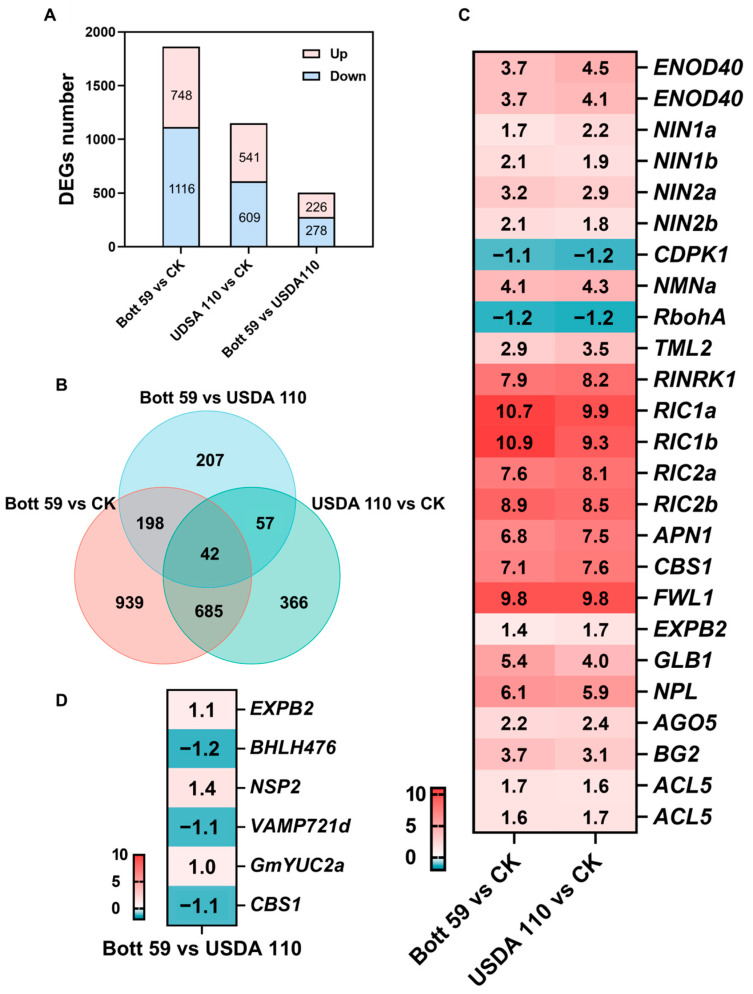
Comprehensive analysis of DEGs across comparisons. (**A**) Statistical summary of DEGs. (|log_2_FC| > 1, *p* < 0.05) in each comparison: Bott 59 vs. CK, USDA 110 vs. CK, and Bott 59 vs. USDA 110. (**B**) Venn diagram illustrating overlap of DEGs between Bott 59 vs. CK, USDA 110 vs. CK comparisons, and Bott 59 vs. USDA 110. (**C**) Heatmap of shared DEGs involved in core symbiotic nitrogen fixation (SNF) in Bott 59 vs. CK and USDA 110 vs. CK comparison groups. (**D**) Heatmap of DEGs related to core SNF in Bott 59 vs. USDA 110 comparison.

To elucidate the molecular similarities and differences in soybean root responses to inoculation with Bott 59 and USDA 110, root samples were collected at 3 dpi, and uninoculated roots were used as a CK for transcriptome sequencing analysis. Sequencing generated an average of 40.2 M clean reads and 5.99 Gb of clean bases per sample, with Q20 scores > 99.5% and Q30 scores > 97.6% (Table S1). To assess biological variability and sample consistency, Principal Component Analysis (PCA) was performed. As shown in Supplementary Figure S1, the three biological replicates for each treatment group (CK, Bott 59, and USDA 110) cluster tightly together, suggesting high experimental reproducibility and low biological noise. Furthermore, the distinct separation between the control and inoculated groups validates that the observed transcriptional changes are driven by the rhizobial treatments rather than random variation. Analysis of DEGs with a threshold of |log_2_ fold change (FC)| > 1 and *p* < 0.05 revealed that, compared to CK, the Bott 59-inoculated group contained 1864 DEGs (748 up-regulated and 1116 down-regulated) (Table S3). In the USDA 110-treated group, 1150 DEGs were identified (541 up-regulated and 609 down-regulated) (Figure 2A) (Table S4). Integrative analysis of the three sample groups identified 727 DEGs (Figure 2B) (Tables S3–S5).

Further analysis of these 727 conserved DEGs revealed a series of established key genes involved in SNF, including the master regulator *NIN*, the early NF signaling component *RINRK1*, nodule primordium development genes *ENOD40* and *NPL*, and core AON pathway genes *RIC* and *TML* [10] (Figure 2C). Additionally, the comparison between Bott 59 and USDA 110 (Figure 2D) showed that genes previously reported to positively regulate symbiosis, such as *EXPB2* [47], *NSP2* [48], and *YUC2a* [49], were up-regulated in response to Bott 59. Conversely, genes known to negatively regulate nodulation, like *BHLH476* [50] and *CBS1* [51], were down-regulated by Bott 59. These transcriptional findings are highly consistent with the nodulation performance, in which Bott 59 exhibits a more potent capacity for promoting SNF compared to USDA 110.

### 2.3. Functional Enrichment Analysis of Similarities and Differences in DEGs Regulated by Two Rhizobial Strains

**Figure 3:**
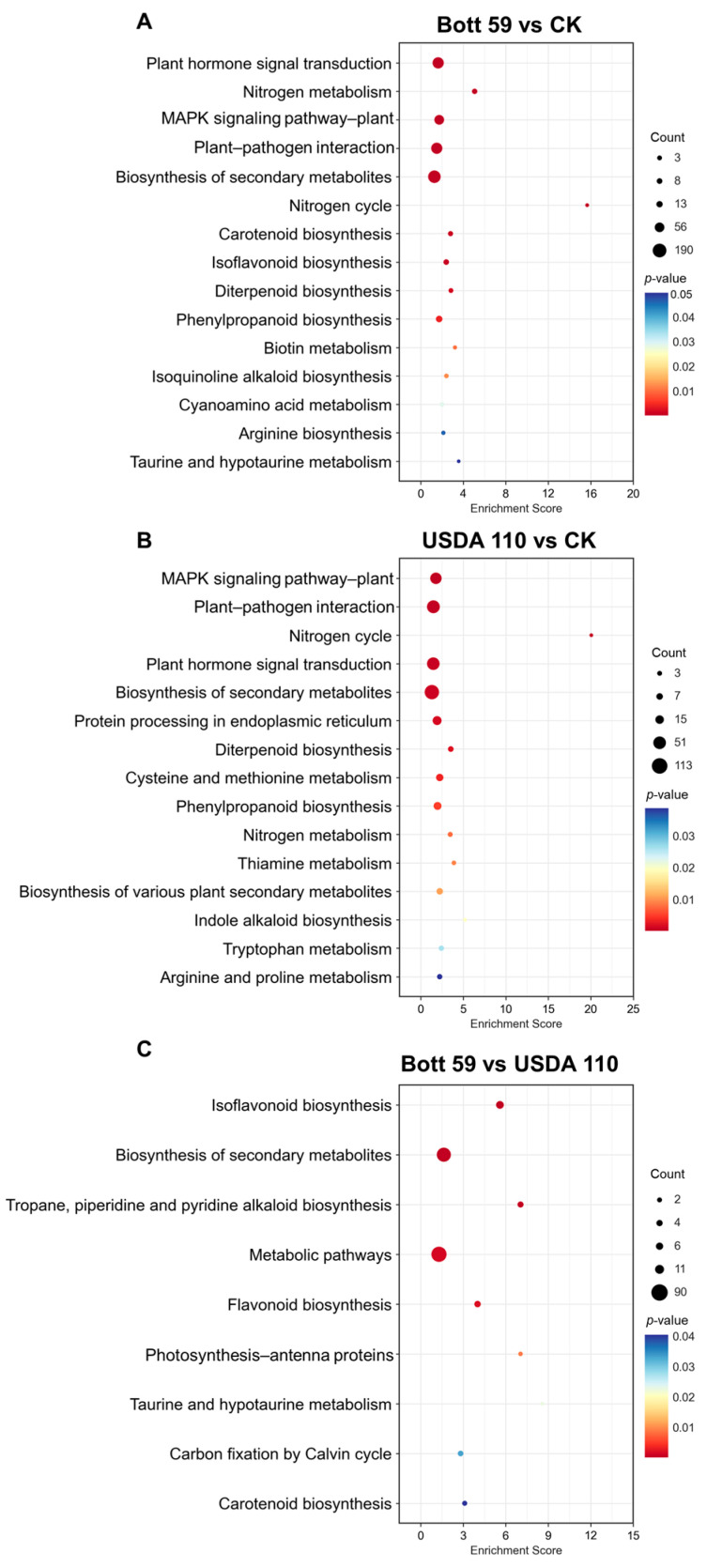
KEGG pathway enrichment analysis of DEGs in root at 3 dpi with rhizobia. (**A**) Comparison between Bott 59 inoculation and CK (Bott 59 vs. CK). (**B**) Comparison between USDA 110 inoculation and CK (USDA 110 vs. CK). (**C**) Comparison between Bott 59 and USDA 110 inoculations (Bott 59 vs. USDA 110). The x-axis represents the Enrichment Score, indicating the degree of pathway enrichment. The size of the bubbles corresponds to the number of DEGs (Count) enriched in each pathway, with larger bubbles indicating a higher gene count. The color gradient represents the *p*-value, ranging from red to blue.

To determine the functional similarity and differences among the DEGs identified, we performed KEGG pathway enrichment analysis (Tables S10–S12). DEGs regulated by both strains were enriched in “Plant hormone signal transduction pathway” and “MAPK signaling pathway” (Figure 3A,B), indicating that the perception of symbiotic signals and the initiation of intracellular signaling cascades are central to the early root response. Although the overall composition of the enriched pathways was similar, Bott 59 regulated a greater number of genes and showed a much stronger induction intensity in the “Plant hormone signal transduction” pathway compared to USDA 110 (Figure 3A,B). This suggests that Bott 59 triggers a more extensive and profound early physiological response in the roots. Further comparison between Bott 59 and USDA 110 (Figure 3C) revealed that DEGs were predominantly enriched in the “Flavonoid biosynthesis” and “Isoflavonoid biosynthesis” pathways.

**Figure 4:**
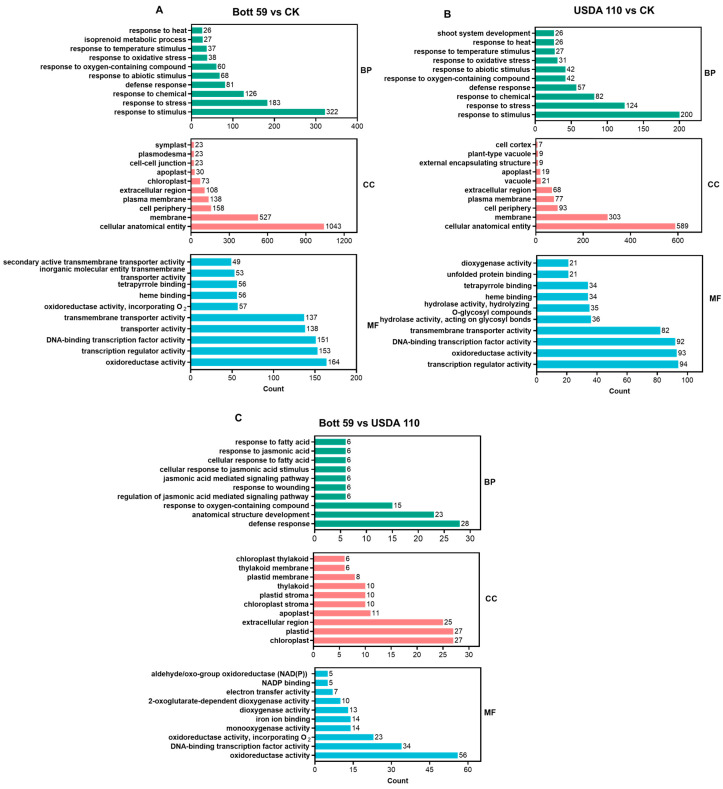
GO functional Enrichment analysis of DEGs in roots at 3 days post-inoculation (dpi) with rhizobia. (**A**) Comparison between Bott 59 inoculation and CK (Bott 59 vs. CK). (**B**) Comparison between USDA 110 inoculation and CK (USDA 110 vs. CK). (**C**) Comparison between Bott 59 and USDA 110 inoculations (Bott 59 vs. USDA 110). The bars are categorized into three Gene Ontology domains: Biological Process (BP), Cellular Component (CC), and Molecular Function (MF). The x-axis represents the number of DEGs enriched in each GO term (Count), and the y-axis lists the specific GO terms. Different colors indicate different ontology categories (BP in green, CC in red, MF in blue).

The root response characteristics were further analyzed using Gene Ontology (GO) enrichment across three categories: Biological Process (BP), Cellular Component (CC), and Molecular Function (MF) (Figure 4) (Tables S13–S15). For BP, Bott 59 vs. CK and USDA 110 vs. CK exhibited high functional conservation, sharing nine core enriched terms (Figure 4A,B). Both strains were strongly induced under “response to stimulus,” “response to stress,” and “defense response,” indicating that signal perception and the initiation of basal defense are the primary physiological responses during early nodulation. Notably, in the Bott 59 vs. USDA 110 comparison, DEGs were enriched in JA-related signaling pathways (Figure 4C), suggesting that Bott 59 possesses a unique signaling mechanism for regulating endogenous hormones and balancing nodulation with defense responses compared to the model strain USDA 110.

In the MF category, conserved terms between Bott 59 and USDA 110 (six terms) focused on “oxidoreductase activity” and “DNA-binding transcription factor activity.” (Figure 4A,B). This indicates that rhizobial infection triggers intense redox balance regulation and large-scale transcriptional reprogramming of downstream genes. Interestingly, the Bott 59 vs. CK comparison indicated unique enrichment in “transporter activity” (Figure 4A,B), implying that Bott 59 may more profoundly influence transmembrane material exchange. In the two-strain comparison, “oxidoreductase activity” and “DNA-binding transcription factor activity” remained dominant, reaffirming inherent differences in transcriptional regulation between the two strains (Figure 4C).

The CC category revealed adaptation mechanisms at the cellular structural level. Both Bott 59 vs. CK and USDA 110 vs. CK comparisons showed significant enrichment in “cell periphery,” “extracellular region,” and “external encapsulating structure.” This suggests that degradation and remodeling of the cell wall provide the structural basis for IT extension during response to rhizobia (Figure 4A,B). Compared to USDA 110, Bott 59 induced more pronounced gene enrichment and significance in terms like “plastid” and “extracellular region” (Figure 4C).

In summary, the symbiotic process between soybean and rhizobia involves highly conserved molecular mechanisms, primarily involving basal defense responses, transcriptional regulation, and membrane structure reorganization. These conserved pathways facilitate receptor recognition and kinase cascades during early SNF. Concurrently, compared to the model strain USDA 110, Bott 59 demonstrates a more efficient and precise molecular regulatory potential by more strongly activating plant hormone signal transduction, specifically inducing isoflavonoid and JA-mediated pathways, and significantly enhancing transporter activity and membrane-related responses. These molecular signatures are highly consistent with the superior SNF phenotype of Bott 59 described previously.

### 2.4. Expression Patterns of Key Genes in Hormone Signaling Pathways

**Figure 5:**
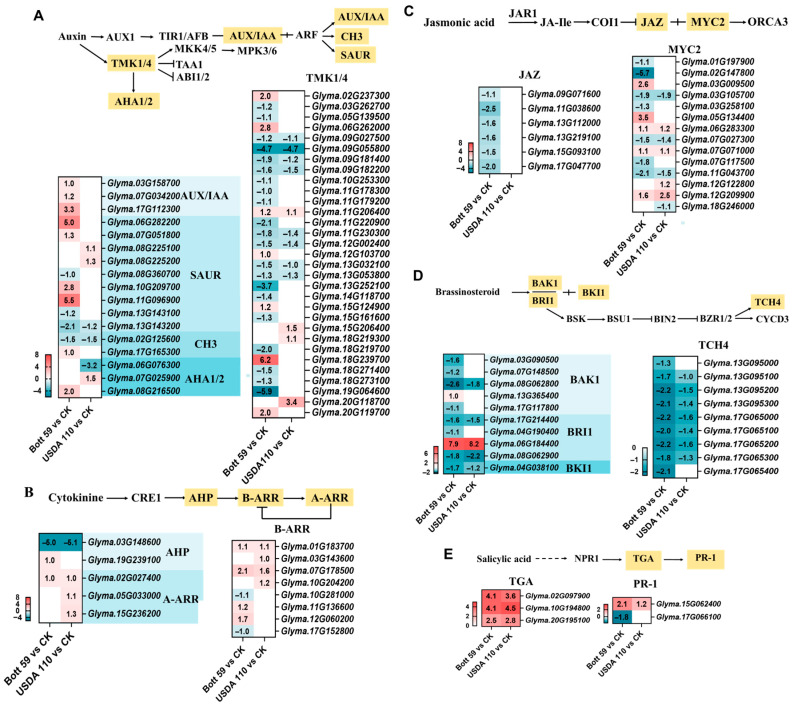
Expression analysis of DEGs involved in auxin (**A**), cytokinin (**B**), brassinosteroid (**C**), jasmonic acid (**D**) and salicylic acid (**E**) signal transduction pathways in soybean roots during early infection by rhizobia. The heatmaps display the log_2_FC values of DEGs. Solid arrows represent direct/confirmed interactions; dashed arrows signify complex, multi-step signaling pathways. Red indicates significant up-regulation, blue indicates significant down-regulation, and blank cells indicate that no significant differential expression was detected in that treatment.

During the early stages of rhizobial infection in legumes, various endogenous hormones respond to coordinate host defense, cellular reprogramming, and the establishment of symbiosis [33]. KEGG enrichment analysis of the 3 dpi transcriptome data identified that “Plant hormone signal transduction” was one of the key pathways enriched with DEGs (Figure 3). Extensive transcriptional regulation across multiple hormone pathways was observed at 3 dpi with either strain (Figure 5). In the auxin signaling pathway, Bott 59 induced a greater number of DEGs compared to USDA 110 (Figure 5A). Specifically, *AUX/IAA* genes, encoding negative regulators of the signaling pathway, were uniquely and up-regulated by Bott 59. Multiple *SAUR* genes were also up-regulated by Bott 59, whereas USDA 110 induced a more moderate response in this family. Differential expression was also observed for *GH3* genes; for example, *Glyma.17G165300* showed an up-regulation pattern, while *Glyma.02G125600* was suppressed in both treatments. As core components of auxin sensing, *TMK1/4*-related genes generally exhibited a down-regulation pattern following inoculation with either strain. Furthermore, genes encoding plasma membrane H^+^-ATPase (*AHA1/2*), which are functionally linked to auxin signaling, were also regulated: *Glyma.08G216500* was up-regulated in Bott 59 (log_2_FC = 2.0), whereas *Glyma.06G076300* was down-regulated in USDA 110 (log_2_FC = −3.2).

Regarding the cytokinin signaling pathway, which provides core signals for triggering nodulation, the gene *Glyma.03G148600* encoding the phosphotransfer protein AHP was down-regulated in both groups (log_2_FC < −5.0). However, another *AHP* homolog, *Glyma.19G239100*, was specifically up-regulated only in the Bott 59 treatment (Figure 5B). The transcriptional response of the core element *B-ARR* was more complex in the Bott 59 treatment (four genes up-regulated, two down-regulated), while all four induced genes in the USDA 110 group were up-regulated. Members of the A-ARR family showed consistent up-regulation following inoculation, though Bott 59 induced only one gene compared to three in USDA 110.

Following inoculation with both strains, the BR signal transduction pathway exhibited highly consistent and synchronous gene expression patterns (Figure 5C). Most homologs of the co-receptor BAK1 were down-regulated by both treatments. Notably, both strains strongly induced the expression of a *BRI1* gene (*Glyma.06G184400*) with log_2_FC values of 7.9–8.2. BKI1 (*Glyma.04G038100*), acting as a “brake” on BR signaling, was synergistically down-regulated in both groups. At the end of the pathway, the TCH4 gene family was consistently down-regulated, with the magnitude of down-regulation being more pronounced in the Bott 59 group. These results indicate that the regulatory mechanisms of BR signaling are highly conserved across different rhizobial strains.

In the JA pathway, *JAZ* (encoding signal repressors) exhibited strain-specific responses: six *JAZ* homologs were down-regulated by Bott 59, whereas no DEGs were detected in the USDA 110 group (Figure 5D). In contrast to *JAZ*, eight *MYC2*-related genes showed almost identical trends and log_2_FC values across both strains. Additionally, Bott 59 uniquely regulated several *MYC2*-related genes, such as *Glyma.02G147800* (log_2_FC = −5.7) and *Glyma.05G134400* (log_2_FC = 3.5), which remained unaffected by USDA 110, indicating that Bott 59 triggers a broader and more intense remodeling of the JA pathway, from upstream JAZ repression to downstream *MYC2* regulation. This divergence suggests that Bott 59 may provide a stronger signal for inducing plant defense or symbiotic regulation than USDA 110. Unlike the differential response of the JAZ module in the JA pathway, the SA pathway showed nearly identical activation patterns for both the TGA and PR-1 modules under both rhizobial treatments (Figure 5E).

Transcriptomic data displayed consistent regulatory directions across multiple hormone signaling pathways following inoculation with different strains, confirming that hormonal regulation is a conserved and essential strategy for establishing symbiosis. While the activation of BR and SA signaling represents a highly conserved molecular mechanism, the auxin, cytokinin, and JA pathways showed distinct strain-specific responses, with Bott 59 inducing more extensive transcriptional reprogramming.

### 2.5. Differential Expression of Key Genes in Isoflavonoid-Related Pathways

**Figure 6:**
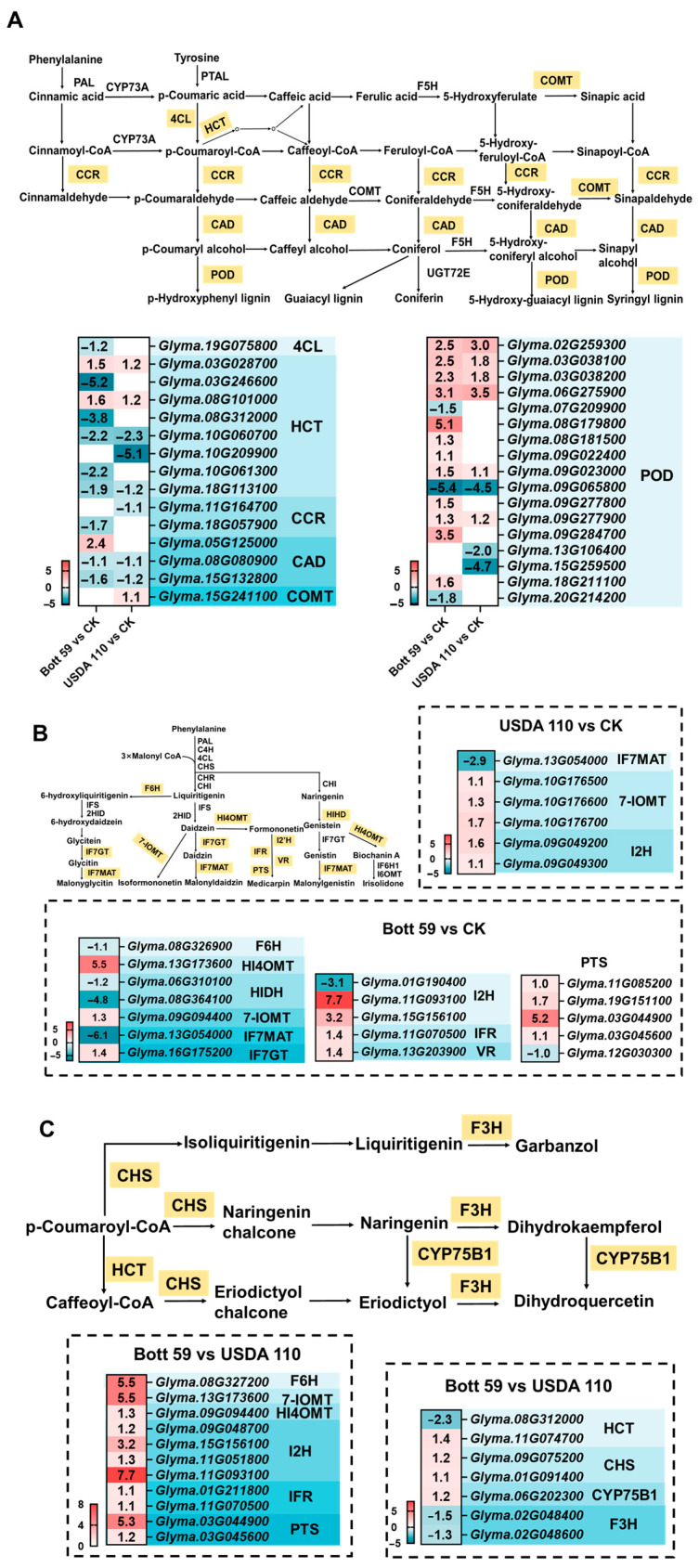
Expression analysis of DEGs from isoflavonoid-related pathways in soybean roots during early rhizobial infection. (**A**) Expression patterns of DEGs in the phenylpropanoid biosynthesis pathway. The heatmap shows DEGs in Bott 59 vs. CK (left columns) and USDA 110 vs. CK (right columns). (**B**) Expression patterns of DEGs in the isoflavonoid biosynthesis pathway for Bott 59 vs. CK. The main heatmap shows DEGs enriched in the Bott 59 vs. CK comparison. The dashed box in the upper right corner displays the corresponding gene responses under USDA 110 treatment. (**C**) Comparative analysis of DEGs in flavonoid biosynthesis pathways between Bott 59 and USDA 110. Values in heatmaps represent log_2_FC. Red indicates significant up-regulation, and blue indicates significant down-regulation. Solid arrows represent direct/confirmed interactions.

Isoflavonoids function as “starter keys” that trigger the molecular dialog between soybean and rhizobia. These early chemical signals induce NF production in rhizobia, initiating the SNF program. Given this central role, understanding the molecular mechanisms of isoflavonoids is of paramount importance [52]. KEGG enrichment analysis showed that both rhizobial strains enriched the “Phenylpropanoid biosynthesis” pathway (Figure 3). Within this pathway, a gene encoding 4CL (*Glyma.19G075800*) was down-regulated only under Bott 59 treatment, while a *COMT* gene (*Glyma.15G241100*) was up-regulated only by USDA 110. Other genes encoding key enzymes, such as *HCT*, *CCR*, and *CAD*, showed largely consistent expression trends across both treatment groups (Figure 6A). The peroxidase (POD) module exhibited extensive and intense transcriptional fluctuations, particularly *Glyma.08G179800*, which was extremely up-regulated by Bott 59 (log_2_FC = 5.1), whereas the induction of this family by USDA 110 was relatively limited. Notably, multiple POD-encoding genes showed consistent induction or suppression trends under both treatments, suggesting the existence of a conserved regulatory mechanism during the early stages of nodulation.

In contrast to the conservation observed in the phenylpropanoid pathway, the isoflavonoid biosynthesis pathway displayed a marked strain-specific preference. This pathway was enriched only in the Bott 59 treatment, with multiple key node genes being activated (Figure 6B). For instance, the log_2_FC values for *HI4OMT* (*Glyma.13G173600*) and *I2H* (*Glyma.11G093100*) reached 5.5 and 7.7, respectively. Additionally, DEGs encoding IFR, VR, and PTS were predominantly activated by Bott 59. Conversely, this pathway did not reach a level of significant enrichment in the USDA 110 group, where the number of related DEGs was sparse and their expression magnitudes were moderate (Figure 6B, top right corner). These results indicate that Bott 59 possesses a superior capacity for inducing isoflavonoid biosynthesis.

Direct comparison between Bott 59 and USDA 110 identified significantly enriched pathways, including the “Isoflavonoid biosynthesis” and “Flavonoid biosynthesis” pathways (Figure 3C). The expression of genes induced by Bott 59, such as *F6H* (*Glyma.08G327200*), *7-IOMT* (*Glyma.13G173600*), *I2H* (*Glyma.11G093100*), and *PTS* (*Glyma.03G044900*), was substantially higher than in the USDA 110 group, with the log_2_FC values for six enzyme-encoding genes ranging from 1.2 to 7.7 (Figure 6C). In the flavonoid biosynthesis pathway, two *CHS* homologs and *CYP75B1* (*Glyma.06G202300*) were up-regulated in the Bott 59 group compared to USDA 110. However, two homologs encoding *F3H* were down-regulated in response to Bott 59 (Figure 6C). This suggests Bott 59 modulates metabolic partitioning, by shunting naringenin, a key branch-point substrate, from flavonoid synthesis toward isoflavonoid biosynthesis.

In conclusion, while the two rhizobial strains induce a relatively conserved transcriptional response in the phenylpropanoid pathway, they exhibit marked strain-specificity in downstream branch pathways. Bott 59 uniquely activates the isoflavonoid biosynthesis pathway and, by potentially inhibiting the flavonoid branch enzyme F3H, may redirect the common substrate naringenin towards isoflavonoids. In contrast, USDA 110 shows minimal induction of this pathway, suggesting a fundamental difference between the two strains in their regulation of flavonoid-related secondary metabolism.

### 2.6. Expression Patterns of Differentially Expressed Transcription Factors (DETFs) in Early Roots Under Incubation Different Rhizobial Strains

Transcriptomic analysis of soybean roots at 3 dpi revealed that GO enrichment results highlighting the pivotal role of transcription factors (TFs) as signaling hubs that coordinate plant defense and symbiotic development (Figure 4). Compared to the control, Bott 59 and USDA 110 inoculation exhibited 200 and 126 significantly differentially expressed transcription factors (DETFs, *p* < 0.05), respectively. A total of 41 DETFs were identified in the Bott 59 vs. USDA 110 comparison (Tables S6–S8).

**Figure 7:**
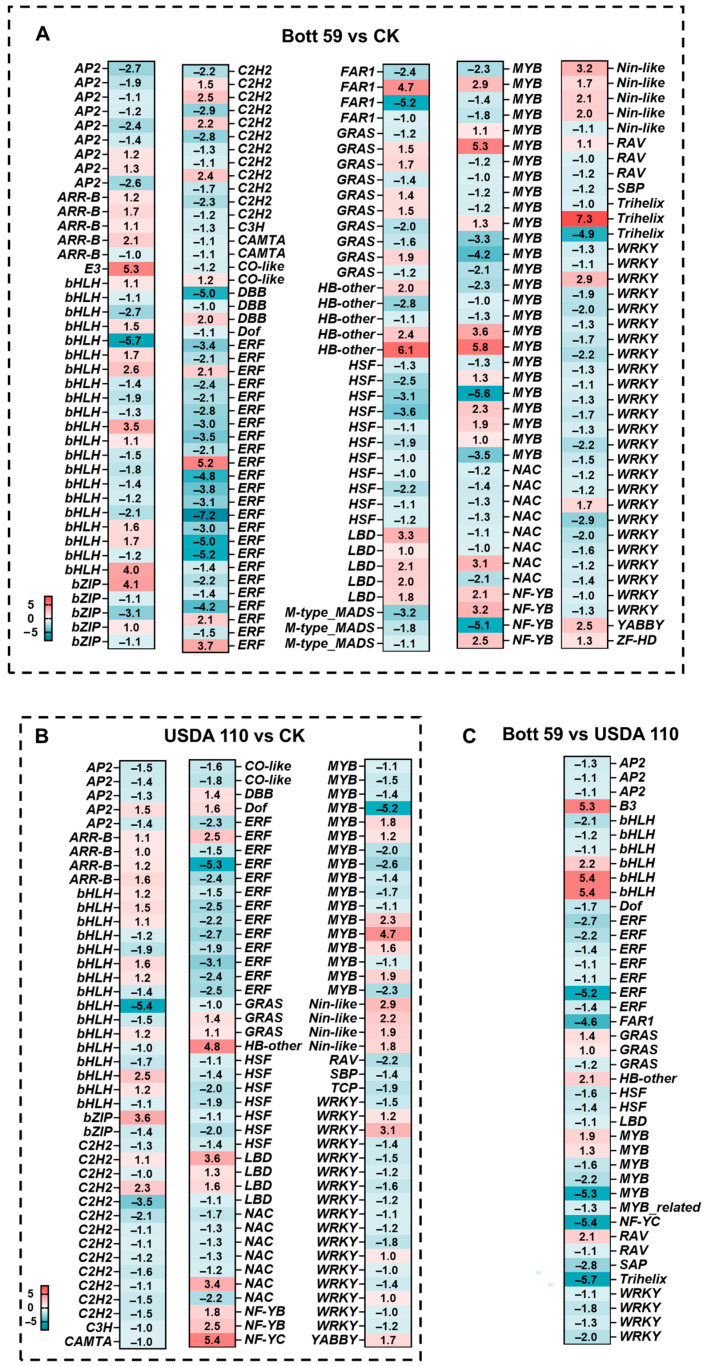
Analysis of DETFs in soybean roots after inoculation with rhizobia. (**A**) Heatmap of DETFs for Bott 59 vs. CK. (**B**) Heatmap of DETFs for USDA 110 vs. CK. (**C**) Comparison of DETFs between Bott 59 and USDA 110. Values in heatmaps represent log_2_FC; color scale from blue (down-regulation) to red (up-regulation).

Following inoculation with these strains, the roots underwent large-scale transcriptional reprogramming involving several core TF families, including AP2, bHLH, ERF, MYB, NAC, NIN-like, and WRKY. Notably, the number of TFs regulated by Bott 59 was generally higher than the number of TFs regulated by USDA 110 (Figure 7A,B). Compared to the CK, both rhizobial strains exhibited conserved expression patterns: AP2, C2H2, ERF, HSF, MYB, NAC, and WRKY members were generally down-regulated, while ARR-B, NIN-like, and LBD members were up-regulated. All four NIN homologs showed consistent up-regulation, whereas NAC family members were predominantly down-regulated following inoculation. These TFs play critical roles in the early stages of nodule development and the establishment of symbiosis [53,54,55,56,57]. The bHLH family showed complex regulation patterns. Notably, the overall response of the ERF and WRKY families was weaker in the USDA 110 treatment compared to Bott 59 (Figure 7A,B).

Specifically, three NF-YB genes were up-regulated in the Bott 59 group, while one homolog was strongly repressed (log_2_FC = −5.1). In contrast, USDA 110 strongly induced an NF-YC member (log_2_FC = 5.4) and two NF-YB members. The NF-Y family is typically involved in the regulation of nodule organogenesis. Additionally, numerous MYB members were suppressed by both strains, with a few specifically activated. These activated MYBs could be key candidates driving the transcription of isoflavonoid biosynthetic genes. Notably, the number of regulated MYB factors in the Bott 59 group was 1.53 times that of the USDA 110 group.

Comparison between the two strains further clarified key regulatory differences (Figure 7C). Compared to USDA 110, Bott 59 up-regulated several bHLH (log_2_FC up to 5.4) and B3 (log_2_FC up to 5.3) family genes. The enrichment of these factors may be directly linked to the more intense activation of the JA pathway and secondary metabolism restructuring. Conversely, the expression of most AP2, ERF, MYB, and WRKY members was higher after inoculation with USDA 110 than with Bott 59. These results suggest that, compared to USDA 110, Bott 59 recruits a more diverse transcription factor network during early infection. By dynamically suppressing basal defense signals and strongly inducing core symbiotic factors, Bott 59 establishes a more favorable environment for efficient nodulation.

### 2.7. Transcript Level Analysis and mRNA Validation by qRT-PCR

**Figure 8:**
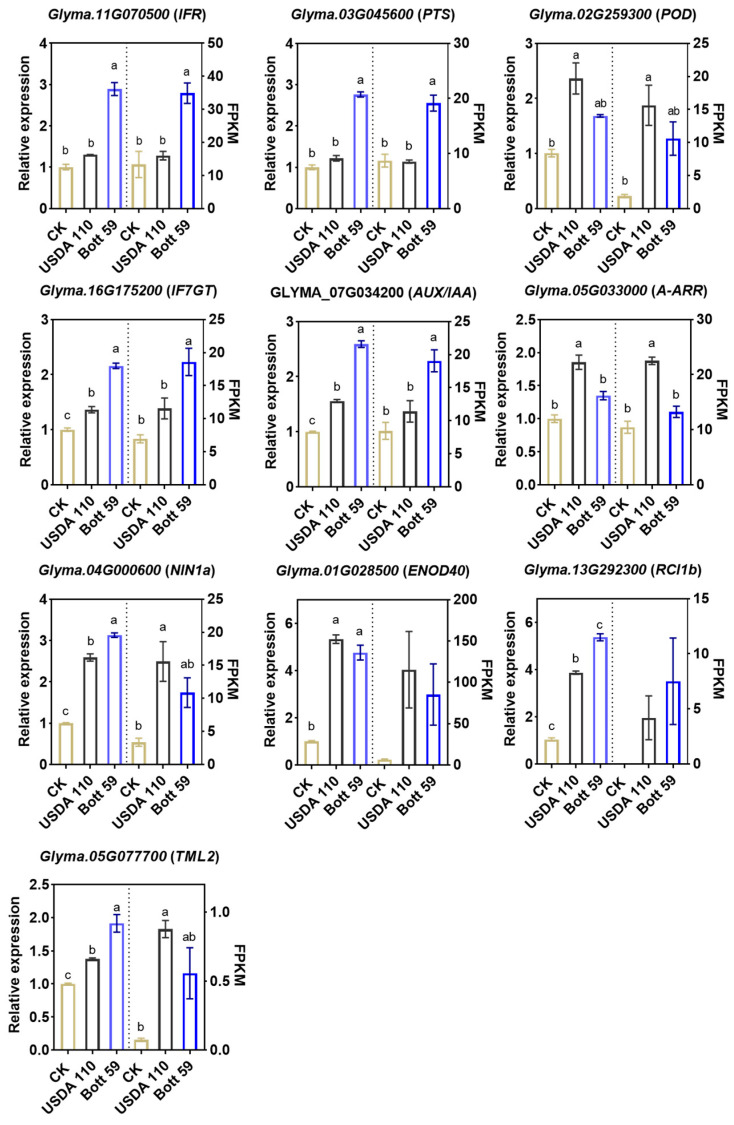
qRT-PCR validation of transcriptomic data. Data are presented as mean ± SE (*n* = 3 independent biological replicates). Different letters indicate statistically significant differences determined by one-way ANOVA followed by Tukey’s multiple comparisons test. All tests were two-tailed, and *p* < 0.05 was considered statistically significant.

To verify the dynamic expression changes in DEGs following infection by rhizobial strains Bott 59 and USDA 110, as well as the robustness of the transcriptomic data, we selected four DEGs involved in the flavonoid signaling pathway (*IFR*, *PTS*, *POD*, and *IF7GT*), genes associated with the hormone transduction signaling pathway (*AUX/IAA* and *A-ARR*), and key SNF genes (*NIN1a*, *ENOD40*, *RIC1b* and *TML2*) for qRT-PCR analysis (Figure 8). The qRT-PCR results exhibited expression trends consistent with the transcriptomic analysis, thereby confirming the accuracy of the RNA-seq data.

## 3. Discussion

Phenotypic evaluations at 21 dpi revealed that the Bott 59-inoculated group exhibited higher nodule numbers, nodule fresh weight, and nitrogenase activity compared to the USDA 110 group. These results indicate that strain Bott 59 enhances SNF efficiency. This suggests the potential for a specific genotype-by-genotype (G × G) interaction [58], wherein the genetic compatibility between the Bott 59 genome and the Williams 82 host underlies enhanced symbiotic efficiency. The molecular basis of such specificity often involves precise recognition mechanisms. For instance, the type III effector NopC in the broad-host-range rhizobium *Sinorhizobium fredii* HH103 affects nodulation across different host genotypes. *NopC* physically interacts with *GmRAC1* to induce the expression of *GmNIN2a/2b* and *GmENOD40*, thereby influencing infection thread extension and nodule primordia initiation [59]. We hypothesize that the exceptional compatibility observed in the W82–Bott 59 pair may result from a unique effector repertoire in Bott 59 that either evades W82-mediated immune surveillance or optimally modulate host signaling pathways, thereby inducing the expression of key symbiosis-related genes in the host. This underscores the critical need to dissect the specific G × G molecular determinants governing this mutualism in future studies.

Furthermore, the enhanced SNF efficiency in the Williams 82–Bott 59 combination may be associated with improvements in host growth, with higher shoot and root biomass compared to inoculation with USDA 110. This aligns with the finding that inoculation with compatible, high-efficiency strains significantly boosts shoot biomass, carbon accumulation, and relative symbiotic effectiveness [60]. The correlation we observed between nodule traits (number and weight) and total plant biomass further supports the notion that efficient G × G matching drives systemic physiological improvements. Ultimately, these biomass gains are likely driven by the synergistic assimilation of carbon through photosynthesis and nitrogen through fixation, a process that is maximized only when the specific genetic requirements of both partners are met [60,61].

Comparative transcriptomic analysis of Bott 59 and USDA 110 provided insights into the early molecular mechanisms underlying enhanced growth-promoting and nitrogen-fixing efficiency of Bott 59. Transcriptomic and functional enrichment analyses showed that after inoculation with either Bott 59 or USDA 110, there were 727 conservatively regulated DEGs, including well-established SNF genes like *NIN*, *RINRK1*, and *ENOD40*, constituting a core transcriptional framework for the soybean response to rhizobia. On the other hand, inoculation with different rhizobial strains (USDA 110, Bott 59) also rapidly activates the AON system and induces significant upregulation of negative regulators such as *RIC* and *TML*, thereby inhibiting excessive nodulation. These results suggest that soybean employs a sophisticated bidirectional regulatory mechanism in response to rhizobial infection. It not only activates signaling pathways related to symbiotic establishment to ensure effective nodulation, but also utilizes the conserved AON system to restrict excessive nodulation in a timely manner, preventing excessive energy and resource expenditure by the host due to overnodulation. This synergistic “promotion–inhibition” regulatory mode represents an adaptive strategy evolved by legumes over long-term evolution, which not only ensures SNF efficiency but also maintains the energy balance between host growth and development and the symbiotic process [22]. Furthermore, both strains exhibited highly conserved regulatory patterns in pathways such as phenylpropanoid biosynthesis, brassinosteroid, and the SA pathway. KEGG and GO enrichment analyses demonstrated that the shared enriched pathways and functional terms were primarily concentrated in basal defense responses, transcriptional regulation, and cell membrane structural remodeling. Two typical environmental sensing pathways—”Plant-pathogen interaction” and “MAPK signaling pathway”—were significantly enriched in both groups. This suggests that during the early stages of contact with different rhizobial strains, soybean roots employ a conserved set of recognition receptors and kinase cascades to achieve the initial perception and transduction of rhizobial signals. The phenylpropanoid biosynthesis pathway, which serves as the foundation of secondary metabolism, was enriched in both treatment groups, with the expression trends of key enzyme genes (e.g., *HCT*, *CCR*, and *CAD*) being essentially consistent (Figure 6A). This implies that before establishing efficient nitrogen fixation, soybean must first activate precursor pathways for lignin and flavonoid synthesis, providing the “material foundation” for subsequent specific signal molecules [62]. Furthermore, at the level of hormone metabolism, the strong induction of the brassinosteroid receptor BRI1 coupled with the synergistic down-regulation of BKI1, as well as the synchronized activation of the TGA and PR-1 modules in the SA pathway, highlights a high degree of consistency in early signal transduction, hormonal balance, and basal metabolism. This suggests that when facing different rhizobial strains, soybean may activate a standardized molecular program to set the tone for the symbiotic relationship, laying the foundation for subsequent strain-specific differential regulation.

Despite this conserved foundation, significant differences were observed. Bott 59 induced approximately 62% more DEGs than USDA 110 (Figure 2). The core of this divergence likely lies in the repartitioning of secondary metabolic signaling. Our results indicate significant differences in the regulation of the isoflavonoid biosynthesis pathway between Bott 59 and USDA 110, which serves as a primary driver for the observed differences in symbiotic efficiency. Bott 59 significantly activated the expression of multiple key nodal genes within the isoflavonoid biosynthesis pathway (Figure 6). As the earliest signals in the molecular dialog between legumes and rhizobia, isoflavonoids (such as daidzein and genistein) stimulate symbiotic nodule signaling and play a critical role in nodule development and nitrogen fixation.

Specifically, Bott 59 appears to enhance symbiotic signal intensity by inhibiting F3H, a key enzyme in the flavonoid biosynthetic branch, thereby shunting more of the common substrate naringenin toward the isoflavonoid pathway. At 3 dpi, during the critical period for nodule primordium initiation [63], Bott 59 induced more pronounced fluctuations in the auxin and JA pathways compared to USDA 110. Since local auxin accumulation at infection sites is a prerequisite for the division of nodule primordia [64]. The greater number of up-regulated *SAUR* induced by Bott 59 suggests enhanced potential for cell elongation and division [65] (Figure 5). The specific downregulation of multiple JAZ repressors in the JA pathway in Bott 59 leads to de-repression of JA signaling, potentially facilitating the trade-off between symbiosis and host immunity. Furthermore, the specific upregulation of an *AHP* gene (*Glyma.19G239100*) in the cytokinin pathway by Bott 59, combined with generally lower levels of *A-ARR* inhibition, may establish a more favorable cytokinin response environment early in nodulation, potentially enhancing cortical cell sensitivity to division signals [66]. This synergistic multi-hormonal regulatory model provides the necessary physiological momentum for the increased nodule production induced by Bott 59 [34,38].

Multiple TF families, including ERF, MYB, NAC, bHLH, and ARF, are known to participate in various nodulation stages, from rhizobial infection to the maintenance of mature nodule function [67]. This study found that despite their different infection efficiencies, both strains consistently induced four *NIN* homologs. NIN is a master coordinator of IT formation and nodule primordium development [19,68], and this conserved response pattern constitutes the foundation of the shared symbiotic regulatory network between the two strains. However, as core components co-regulating nodule organogenesis with NIN, the NF-Y family exhibited intriguing differences between the two treatments: Bott 59 primarily drove development through the significant up-regulation of three NF-YB members, whereas USDA 110 induced extremely intense transcriptional fluctuations in NF-YC members. Since the NF-YC1 subunit controls not only infection and nodule organogenesis but also contributes to strain-specific rhizobial selection [69], this differential preference for NF-Y subunits could influence final nodule numbers. MYB TFs are key regulators of phenylpropanoid and isoflavonoid metabolism [70]. The greater number of regulated MYB DETFs in the Bott 59 treatment aligns perfectly with the strong induction of isoflavonoid synthesis observed. Notably, the two-strain comparison showed that Bott 59 upregulated several bHLH and B3 family genes (Figure 7C). Differential responses of bHLH and B3 families can drive secondary metabolism and hormone signal remodeling [62]. B3 family members are often coupled with auxin and JA signaling [71], potentially participating in the more intense JA pathway remodeling during Bott 59 infection, thereby conferring superior symbiotic adaptability to Bott 59. This study also found that the AP2, ERF, and WRKY families exhibited distinct expression patterns under the two strain treatments. In the USDA 110 treatment, the expression levels of these TFs, which are typically associated with plant basal immunity (PTI), were higher than those in Bott 59 (Figure 7C). This suggests that during USDA 110 infection, soybean roots may maintain a higher state of defensive alertness. In contrast, Bott 59 appears to better coordinate TF expression to effectively suppress the host’s basal defense at the onset of symbiosis, thereby reducing colonization resistance.

The genes specifically and highly induced by Bott 59 identified in this study represent potential targets for molecular improvement of soybean nodulation efficiency. Two core genes in the isoflavonoid pathway, *HI4OMT* and *I2H*, are prime candidates for enhancing the intensity of symbiotic signaling. The hyper-activation of these two genes by Bott 59 suggests that it constructs a molecular dialog signal in establishing symbiosis with soybean that far exceeds the magnitude of that produced by USDA 110. Furthermore, *AHP* (*Glyma.19G239100*) demonstrated distinct strain selectivity, being significantly up-regulated only after Bott 59 inoculation. Serving as a “messenger” that transmits cytokinin signals from plasma membrane receptors to the nucleus, the expression level of AHP directly influences the sensitivity of cortical cells to mitotic (division) signals. Targeted expression of this *AHP* homolog, driven by root-specific promoters, could precisely enhance the sensitivity of cortical cells in perceiving symbiotic signals. In summary, these genes (e.g., *HI4OMT*, *I2H*, and *AHP*) that exhibit extreme induction characteristics in Bott 59 are not only the molecular keys to elucidating the high-efficiency growth-promotion mechanism of Bott 59 but also represent vital genetic resources for future precision breeding. Utilizing CRISPR/Cas9 or tissue-specific overexpression technologies to modify these targets holds the promise of developing a new generation of soybean varieties characterized by the “autonomous selection of high-efficiency strains,” thereby significantly increasing the contribution rate of biological nitrogen fixation in agricultural production.

In this study, we systematically profiled the transcriptional responses of host plants at 3 dpi with diverse rhizobial strains, and identified conserved and strain-specific patterns of transcriptional regulation, including those of genes involved in multiple metabolic pathways. These findings provide essential data support for further dissecting the molecular mechanisms underlying soybean–rhizobium SNF. However, relying solely on single time-point data makes it difficult to comprehensively depict the continuous dynamic processes of nodule morphogenesis and functional differentiation. Meanwhile, whether such differentially regulated transcriptional changes can be directly translated into distinct alterations in host metabolic metabolites still requires systematic validation and clarification. Collectively, future studies employing diverse rhizobial strains, spanning the full symbiotic continuum from early infection to nodule senescence, and performing integrated multi-omics analyses complemented by metabolomics will provide more comprehensive datasets for systematically elucidating the molecular basis and metabolic regulatory mechanisms underlying strain-specific symbiotic interactions.

## 4. Materials and Methods

### 4.1. Plant and Rhizobial Culture

Seeds of soybean (*Glycine max* L. Merr.) cv. Williams 82 were sterilized by immersion in 75% ethanol and 3% H_2_O_2_ for 5 min with gentle agitation, followed by at least five washes with sterile ddH_2_O. Sterilized seeds were germinated in sterile vermiculite and irrigated with a low-nitrogen nutrient solution. The B & D nutrient solution [72] composition was as follows: 1000 μM CaCl_2_·2H_2_O, 500 μM KH_2_PO_4_, 10 μM Fe-citrate, 250 μM MgSO_4_·7H_2_O, 1500 μM K_2_SO_4_, 1 μM MnSO_4_, 2 μM H_3_BO_4_, 0.5 μM ZnSO_4_·7H_2_O, 0.2 μM CuSO_4_·5H_2_O, 0.1 μM CoSO_4_·7H_2_O, 0.1 μM Na_2_MoO_4_·2H_2_O, and 0.25 mM KNO_3_. The nutrient solution was prepared with deionized water, and the pH was adjusted to 6.8. Soybean plants were maintained in a controlled growth chamber at 26 °C with a 16 h light/8 h dark photoperiod, a light intensity of 200 μmol/m^2^/s, and 60% relative humidity.

The rhizobial strains used in this study were *B. ottawaense* Bott 59 and *B. diazoefficiens* USDA 110. Strains were cultured in TY liquid medium (5.0 g/L tryptone, 3.0 g/L yeast extract, and 0.9 g/L CaCl_2_·2H_2_O; sterilized at 121 °C for 20 min). Cultures were grown at 28 °C with shaking at 200 rpm on a rotary shaker. Once the culture reached an optical density at 600 nm (OD_600_) of approximately 0.8, cells were harvested by centrifugation (4000× *g*, 10 min, 4 °C). The supernatant was discarded, and the cell pellet was resuspended in sterile water, adjusting the OD_600_ to 0.05. Ten-day-old soybean seedlings were inoculated by applying 20 mL of the rhizobial suspension to the root zone of each plant. Control plants were treated with an equal volume of sterile water.

For RNA-Seq analysis, root tissues (approximately 1.5 cm from the root base, with root tips removed) were collected at 3 dpi. To minimize transcriptomic fluctuations derived from stochastic plant-to-plant variation and microenvironmental noise, while retaining inherent biological variance across replicates, we established three independent biological replicates, with each sample generated by pooling root tissues from four individual plants.

### 4.2. RNA Extraction and Library Construction

Total RNA was extracted from root samples using the R6834 Total RNA Kit I (Omega Bio-tek, Norcross, GA, USA). RNA concentration and purity were assessed using a NanoDrop One spectrophotometer (NanoDrop Technologies, Wilmington, DE, USA), and precise quantification was performed using a Qubit 3.0 Fluorometer (Life Technologies, Carlsbad, CA, USA). RNA integrity was further verified via 1.0% agarose gel electrophoresis. Sequencing libraries were prepared using the MGIEasy RNA Library Prep Kit (MGI Tech, Shenzhen, China). Following quality control, the libraries were sequenced on the DNBSEQ-T7 platform (MGI Tech, Shenzhen, China) using a 150 bp paired-end (PE150) strategy.

### 4.3. Data Processing and Functional Enrichment Analysis of DEGs

Raw sequencing data were filtered using fastp (v0.21.0) to obtain clean reads. The high-quality reads were then aligned to the soybean reference genome (Glycine max Wm82.a2.v1, https://plants.ensembl.org/Glycine_max/Info/Index, accessed on 6 January 2026) using HISAT2 (2.2.1). Raw count data were normalized using the median of ratios method to account for differences in library size and RNA composition. Gene expression levels were quantified using RSEM software (1.3.3) and are reported in FPKM (Fragments Per Kilobase of transcript per Million mapped reads). DEGs were identified using DESeq2 (1.34.0), with potential batch effects statistically corrected by explicitly including the batch variable in the design matrix (design = ~batch + condition). Genes with a *p*-value < 0.05 and |log_2_FC| > 1 were considered significantly differentially expressed.

To characterize the functional roles of the DEGs, GO enrichment analysis (http://geneontology.org/, accessed on 5 February 2026) was performed across three categories: BP, MF, and CC. Pathway enrichment analysis was conducted using the Kyoto Encyclopedia of Genes and Genomes (KEGG) database (https://www.kegg.jp/, accessed on 5 February 2026) via the clusterProfiler R package. Furthermore, transcription factors (TFs) among the DEGs were identified and classified into specific families using the PlantTFDB database (https://planttfdb.gao-lab.org/, accessed on 5 February 2026) based on the criteria of |log_2_FC| > 1 and *p*-value < 0.05.

### 4.4. Nitrogenase Activity Assay

Nitrogenase activity was determined via the Acetylene Reduction Assay (ARA) as described by Liang et al. [73]. Briefly, cleaned root samples were placed in 60 mL reaction vials and sealed. After removing 3.0 mL of air from the vial using a syringe, an equal volume of C_2_H_2_ was immediately injected. The bottles were incubated at 28 °C for 2 h. 5.0 mL of the gas phase was collected in 20 mL Headspace Vials Clear and analyzed using a Shimadzu GC2030 gas chromatograph equipped with FID and an HS-10 automatic headspace sampler (Shimadzu, Kyoto, Japan). The C_2_H_4_ concentration was quantified based on the peak area of a known standard, and C_2_H_4_ content (μmol) per plant sample was calculated. ARA was calculated by normalizing to nodule fresh weight and expressed as μmol C_2_H_4_·g^−1^·h^−1^.

### 4.5. RT-qPCR Validation

Ten genes were selected for qRT-PCR validation. Primers were designed using primer3Plus (https://www.primer3plus.com, 25 March 2026) (Table S2). *GmACT11* was used as an internal reference gene for normalization of gene expression. Pre-extracted RNA was reverse transcribed into cDNA using a two-step qRT-PCR kit based on hiscript II reverse transcriptase (Vazyme Biotech Co., Ltd., Nanjing, China). Amplification reactions were prepared using ChamQ Universal SYBR qPCR Master Mix (Nanjing Vazyme Biotech Co., Ltd., Nanjing, China), and qRT-PCR was performed using the QuantStudio 5 Real-Time PCR System (Applied Biosystems, Thermo Fisher Scientific, Waltham, MA, USA). Relative gene expression was determined using the 2^−ΔΔCt^ method. Three technical replicates were performed for each gene in the validation.

### 4.6. Statistical Analysis

Statistical analyses were performed using GraphPad Prism 9. Differences between the two groups were evaluated using a paired two-tailed t-test. For multiple comparisons, one-way analysis of variance (ANOVA) test was employed. All data are presented as the Mean ± Standard Error (SE). A *p* < 0.05 was considered statistically significant.

### 4.7. Data Availability Statement

The raw data supporting the conclusions of this article have been deposited at CNCB (https://www.cncb.ac.cn/, 9 April 2026), under Bio Project IDs PRJCA062000.

## 5. Conclusions

This study systematically characterizes the growth traits, symbiotic phenotypes, and early root transcriptomic responses through a comparative analysis of the soybean rhizobial strain Bott 59 and the model strain USDA 110. Compared to USDA 110, Bott 59 enhances plant biomass, nodule number, and nodule weight during its symbiotic association with Williams 82. The elevated ARA underscores the exceptional growth-promoting and nitrogen-fixation potential of Bott 59. Transcriptomic analysis reveals that at 3 dpi, Bott 59 induces 62% more DEGs than USDA 110.

While both strains share conserved mechanisms for mobilizing the common symbiosis signaling pathway (e.g., NIN), phenylpropanoid biosynthesis pathway, brassinosteroid, and SA signal transduction pathway, Bott 59 elicits a broader range of gene responses. Notably, transcriptomic profiling indicates a pronounced enrichment of isoflavonoid biosynthesis-associated genes following Bott 59 inoculation, with upregulation of key pathway genes including *HI4OMT* and *I2H*. These data suggest that Bott 59 effectively reinforces the early molecular dialog with the host, thereby accelerating nodule primordium initiation. Bott 59 elicits more robust auxin, cytokinin, and JA signaling responses while orchestrating a more extensive array of TFs from the MYB, bHLH, and B3 families. This sophisticated regulatory network likely drives the rapid physiological transition of the root system from basal defense to high-efficiency symbiotic development. While these correlations are strong, future functional assays will be essential to definitively dissect the causal mechanisms within this network.

In conclusion, Bott 59 shows great potential for high-efficiency nitrogen fixation. We clarified the molecular mechanisms driving its improved growth performance and pinpointed key regulators, such as *HI4OMT* and *I2H*. Collectively, these results provide valuable insights for enhancing soybean nitrogen fixation via breeding and genetic engineering.

## Data Availability

The original contributions presented in this study are included in the article/Supplementary Materials. Further inquiries can be directed to the corresponding authors.

## Supplementary Materials

The following supporting information can be downloaded at: https://www.mdpi.com/article/10.3390/plants15091417/s1, Table S1: Sample raw data information statistics. Table S2: List of primers for qRT-PCR used in this study. Table S3: Total DEGs (*p*-value < 0.05) in soybean roots: Bott 59-inoculated vs. CK. Table S4: Total DEGs (*p*-value < 0.05) in soybean roots: USDA 110-inoculated vs. CK. Table S5: Total DEGs (*p*-value < 0.05) in soybean roots: Bott 59-inoculated vs. USDA 110-inoculated. Table S6: Total DETFs (*p*-value < 0.05) in soybean roots: Bott 59-inoculated vs. CK. Table S7: Total DETFs (*p*-value < 0.05) in soybean roots: USDA 110-inoculated vs. CK. Table S8: Total DETFs (*p*-value < 0.05) in soybean roots: Bott 59-inoculated vs. USDA 110-inoculated. Table S9: FPKM values of all expressed genes in soybean roots under CK, USDA 110 inoculated, and Bott 59. Table S10: KEGG enrichment analysis of Bott 59-inoculated vs. CK. Table S11: KEGG enrichment analysis of USDA 110-inoculated vs. CK. Table S12: KEGG enrichment analysis of Bott 59-inoculated vs. USDA 110-inoculated. Table S13: GO enrichment analysis of Bott 59-inoculated vs. CK. Table S14: GO enrichment analysis of USDA 110-inoculated vs. CK. Table S15: GO enrichment analysis of Bott 59-inoculated vs. USDA 110-inoculated. Figure S1: PCA analysis of transcriptomic data across the same time points showed that PC1 and PC2 explained 23.33% and 18.05% of the variance, respectively.

## Abbreviations

The following abbreviations are used in this manuscript:

SNF	symbiotic nitrogen fixation
NF	Nod factor
ITs	infection threads
LYKs	LysM-type receptor-like kinases
AON	Autoregulation of Nodulation
PAL	phenylalanine ammonia-lyase
4CL	4-coumarate-CoA ligase
CHS	chalcone synthase
IFS	isoflavone synthase
RNAi	RNA interference
SA	Salicylic acid
JA	jasmonic acid
DEGs	differentially expressed genes
dpi	days post-inoculation
GO	Gene Ontology
BP	Biological Process
MF	Molecular Function
CC	Cellular Component
KEGG	Kyoto Encyclopedia of Genes and Genomes
TFs	transcription factors
ARA	Acetylene Reduction Assay
POD	peroxidase
FPKM	Fragments Per Kilobase of transcript per Million mapped reads
