# Supplementary material for: Transcriptomic Comparison of Soybean Roots Inoculated with Different Rhizobium Strains During Early Symbiosis
Source: Plants (Basel). 2026 May 6;15(9):1417. doi: 10.3390/plants15091417 (PMC13165157; doi:10.3390/plants15091417)
Supplement: Supplementary file 1 [file plants-15-01417-s001.zip › Supplementary figureS1.pdf]

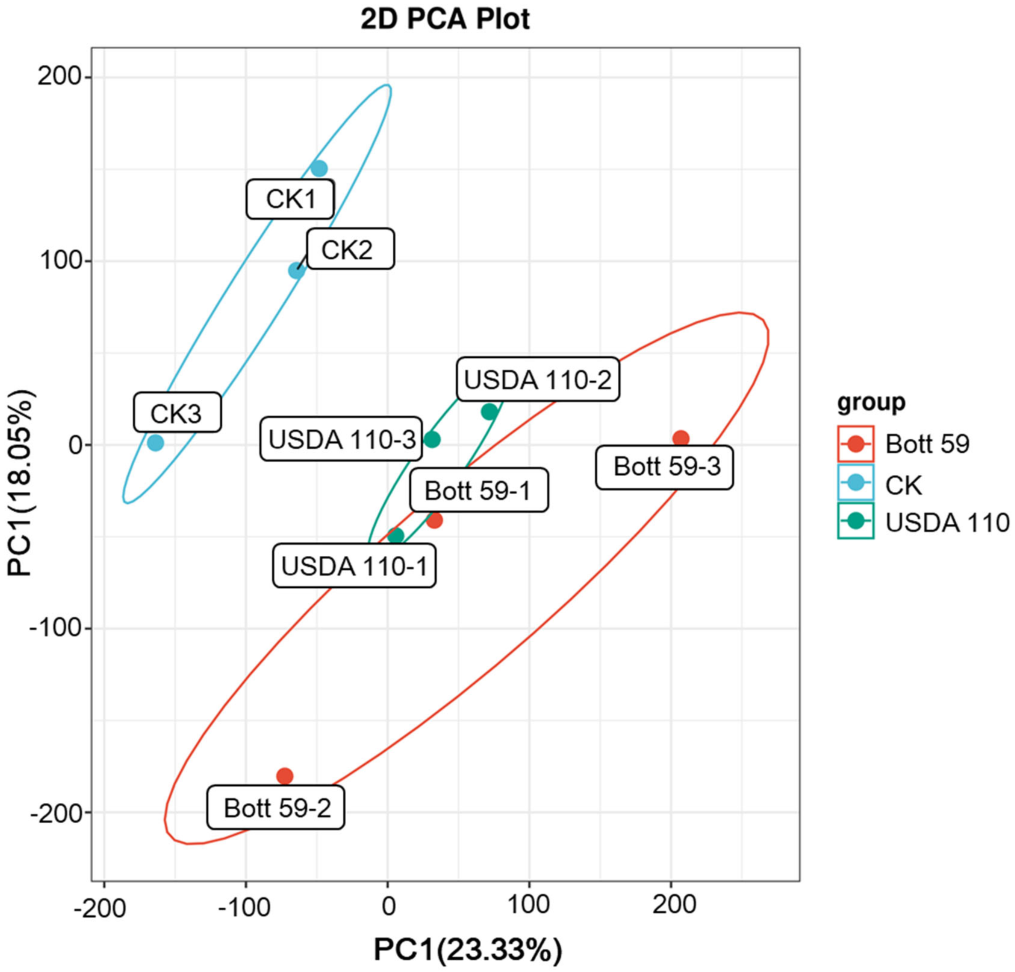

Figure S1. PCA analysis of transcriptomic data across the same time points showed that PC1 and PC2 explained 23.33% and 18.05% of the variance, respectively.
